# Arsenic and Antimony Transporters in Eukaryotes

**DOI:** 10.3390/ijms13033527

**Published:** 2012-03-15

**Authors:** Ewa Maciaszczyk-Dziubinska, Donata Wawrzycka, Robert Wysocki

**Affiliations:** Department of Genetics and Cell Physiology, Institute of Plant Biology, University of Wroclaw, Kanonia 6/8, 50-328 Wroclaw, Poland; E-Mail: donata.wawrzycka@biol.uni.wroc.pl

**Keywords:** arsenic, antimony, aquaglyceroporins, Acr3 antiporter, ABC transporters, glucose transporters, glutathione, phytochelatin

## Abstract

Arsenic and antimony are toxic metalloids, naturally present in the environment and all organisms have developed pathways for their detoxification. The most effective metalloid tolerance systems in eukaryotes include downregulation of metalloid uptake, efflux out of the cell, and complexation with phytochelatin or glutathione followed by sequestration into the vacuole. Understanding of arsenic and antimony transport system is of high importance due to the increasing usage of arsenic-based drugs in the treatment of certain types of cancer and diseases caused by protozoan parasites as well as for the development of bio- and phytoremediation strategies for metalloid polluted areas. However, in contrast to prokaryotes, the knowledge about specific transporters of arsenic and antimony and the mechanisms of metalloid transport in eukaryotes has been very limited for a long time. Here, we review the recent advances in understanding of arsenic and antimony transport pathways in eukaryotes, including a dual role of aquaglyceroporins in uptake and efflux of metalloids, elucidation of arsenic transport mechanism by the yeast Acr3 transporter and its role in arsenic hyperaccumulation in ferns, identification of vacuolar transporters of arsenic-phytochelatin complexes in plants and forms of arsenic substrates recognized by mammalian ABC transporters.

## 1. Introduction

Arsenic and antimony are toxic metalloids which are commonly present in the environment. Arsenic contamination of drinking water and soils from both natural and anthropogenic sources is a major hazard to human health in many areas [1]. It is estimated that more than 40 million people worldwide are chronically exposed to dangerous levels of arsenic which leads to several diseases, including many types of cancer [2]. On the other hand, because of their cytotoxic properties arsenic and antimony compounds are also parts of modern anticancer and antiprotozoan therapies. Arsenic trioxide is routinely used in the clinical treatment of acute promyelocytic leukemia and trialed together with other inorganic (e.g., realgar) and organic arsenicals (e.g., Melarsoprol) in the treatment of hematological malignancies and solid tumors [3]. Importantly, pentavalent antimony-based compounds are still the first-line drugs for the major tropical disease leishmaniasis [4], while the treatment of second neurological stage of human African trypanosomiasis, known as sleeping sickness, has exclusively relied on trivalent arsenic-containing drug Melarsoprol until the recent introduction of eflornithine [5].

Studies on arsenic and antimony transporters have received much attention as these proteins are the key components of both accumulation and detoxification systems for metalloids [6–8]. Last fifteen years of research in this field greatly advanced our comprehension of routes of metalloid uptake, efflux and sequestration into intracellular compartments in various eukaryotes from yeast to humans. This knowledge has significantly contributed to understanding how cancer cells and parasites acquire tolerance to arsenic and antimony-containing drugs and how metalloid resistance can be prevented. In near future it may also help to construct genetically modified crops with low accumulation of arsenic or plant hyperracumulators for remediation of metalloid-polluted areas.

## 2. Pathways for Arsenic and Antimony Uptake

**Figure 1:**
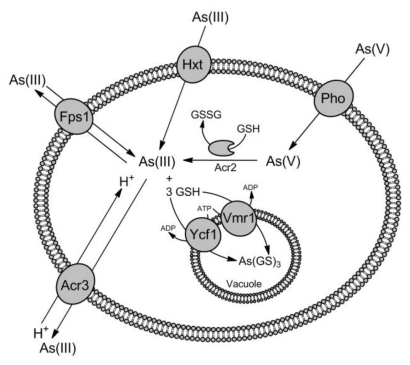
Pathways for arsenic uptake and detoxification in the yeast *S.cerevisiae*. In yeast cells uptake of As(III) is facilitated mainly via the aquaglyceroporin Fps1 but in the absence of glucose As(III) also enters the cell through the hexose permeases Hxt (Hxt1-Hxt17, Gal2). As(V) is taken up by the phosphate transporters (Pho), such as Pho84 and Pho87, followed by reduction to As(III) by the arsenate reductase Acr2 in the cytoplasm. Next, As(III) is transported out of the cell by the As(III)/H^+^ antiporter Acr3 against the concentration gradient or by the aquaglyceroporin Fps1, especially during As(V) exposure, when the internal concentration of As(III) is higher than the outside. In addition, As(III) is conjugated with glutathione (GSH) and sequestrated into the vacuole by the ABC transporters Ycf1 and Vmr1.

**Figure 2:**
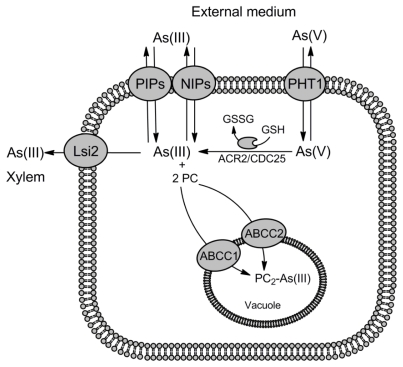
Routes for arsenic transport in higher plants. In plant cells As(III) is accumulated through the aquaporins of Nodulin26-like intrinsic protein subfamily (NIPs) and plasma membrane intrinsic protein subfamily (PIPs), while As(V) uptake is catalyzed by the phosphate transporters (PHT1). In the cytoplasm As(V) is rapidly reduced to As(III) by the arsenate reductase ACR2/CDC25. Upon binding to phytochelatins (PCs), PC_2_-As(III) complexes are compartmentalized into the vacuole by two *A. thaliana* ABC transporters, ABCC1 and ABCC2. As(III) can also leak out of the plant root cell via the NIP and PIP channels down the concentration gradient to external medium. On the other hand, in rice the silicon transporter Lsi2 localizing to the proximal side of root cells extrudes As(III) to the xylem contributing to metalloid accumulation in shoots and grain.

**Figure 3:**
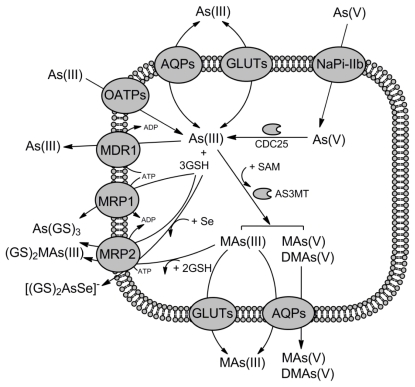
Summary of arsenic transport pathways in mammalian cells. As(V) uptake is mediated by the high-affinity phosphate transporter NaPiIIb. Next, As(V) is reduced to As(III) by CDC25 phosphatases/arsenate reductases. As(III) is transported into the mammalian cells via multiple pathways: the aquaglyceroporins (AQP3, AQP7, AQP9, AQP10), the glucose permeases (GLUT1, GLUT2, GLUT5) or the organic anion transporting polypeptides (OATPB, OATPC). The AQP9 and GLUT2 transporters are also responsible for the transport of methylated species of As(III) out of the cells. The ABC transporters from the ABCB (MDR1/P-gp) and ABCC (MRP1 and MRP2) subfamilies are the major pathways of As(III) extrusion. Both MRP1 and MRP2 are able to transport inorganic and monomethylated forms of As(III) conjugated with glutathione. In addition, MRP2 mediates efflux of seleno-bis(*S*-glutathionyl) arsinium ion. The exact form of As(III) recognized by MDR1/P-gp is uncertain but it is not glutathione-*S*-conjugate.

Arsenic and antimony are highly toxic and can only permeate cells through the transporters evolved for accumulation of essential ions and nutrients using the molecular mimicry. In aqueous solution at the physiological pH, trivalent arsenite [As(III)] and antimonite [Sb(III)] exist mainly in the trihydroxylated uncharged forms, As(OH)_3_ and Sb(OH)_3_, which structurally resemble glycerol [9,10]. Thus, in all domains of life organisms easily accumulate As(III) and Sb(III) by the aquaglyceroporins, a ubiquitous family of membrane proteins, which are permeable for water and glycerol (Figures 1–3) [11,12]. In addition, As(III) and Sb(III) can enter the cells via hexose transporters. Arsenate [As(V)] behaves as a chemical analogue of inorganic phosphate and is taken up by the phosphate transporters (Figures 1–3) [6,7]. In contrast antimonate [Sb(V)] exists as [Sb(OH)_6_]^−^ anion and does not compete with the phosphate uptake [13,14]. The pathway of Sb(V) entry into the cells has not been determined yet.

### 2.1. Uptake of As(V) via Phosphate Transporters

In the yeast *Saccharomyces cerevisiae* two high-affinity, Pho84 and Pho89, and three low-affinity, Pho87, Pho90 and Pho91, phosphate transporters have been identified [15]. Deletion of *PHO84* and *PHO87* genes resulted in increased As(V) tolerance strongly, suggesting that As(V) uptake is mediated by phosphate transport system in yeast (Figure 1) [16,17]. Moreover, cells lacking the membrane protein Pho86, which is required for targeting Pho84 to the plasma membrane, and the phosphate transporter-associated proteins, Gtr1 and Pho88, which regulate positively transport activity of Pho84, also exhibited increased resistance to As(V) [16–18].

Since the As(V)-tolerant plants display constitutive suppression of high-affinity phosphate uptake system [19,20] and As(V) import is inhibited by the presence of phosphate [21], it has been generally accepted that plants accumulate As(V) via the phosphate transporters. However, the involvement of particular phosphate transporters in As(V) intake has not been demonstrated. Recently, based on the genetic data, two phosphate transporters, Pht1;1 and Pht1;4, have been proposed to be responsible for As(V) uptake in *Arabidopsis thaliana* (Figure 2) [22,23]. The *A. thaliana* mutant lacking both Pht1;1 and Pht1;4 exhibited high resistance to As(V) [22]. In addition, mutation in the *A. thaliana PHF1* gene, which blocks Pht1;1 trafficking from the endoplasmic reticulum to the plasma membrane resulted in increased tolerance to As(V) [23]. In a more recent report, it has been presented that the rice Pht1 (OsPht1;8) mediates high-affinity transport of As(V) and transgenic lines overexpressing OsPht1;8 accumulated high-levels of As(V) [24].

Transporters responsible for As(V) uptake has been recently identified in vertebrates. Five rat sodium/phosphate transporters NaPiIIa, NaPiIIb, NaPiIIc, Pit-1 and Pit-2, which constitute the mammalian phosphate uptake system, were expressed in *Xenopus laevis* oocytes to analyze directly transport of radioactive As(V) [25]. NaPiIIa, NaPiIIc, Pit-1 and Pit-2 occurred to have 10-fold lower affinity for As(V) than for inorganic phosphate, suggesting their negligible role in As(V) accumulation. In contrast, NaPiIIb from rat, mouse and human showed high affinity for As(V) and was proposed to be a major entry for As(V) in the intestine (Figure 3) [25]. Similarly, NaPi-IIb1 from zebrafish is also involved in As(V) transport [26].

### 2.2. Aquaglyceroporins Are the Major Cellular Entrance for As(III) and Sb(III)

The first evidence suggesting that the aquaglyceroporins are the entry pathway for metalloids comes from the study of Sanders *et al.* [27] showing that inactivation of *Escherichia coli glpF* gene, encoding for the glycerol facilitator, led to Sb(III) resistance phenotype. Later, based on the genetic data and direct transport measurements of radioactive As(III), Wysocki *et al.* [28] have demonstrated that the *S. cerevisiae* glycerol facilitator Fps1 mediates uptake of As(III) and Sb(III) (Figure 1). Both glycerol facilitators GlpF and Fps1 belong to the family of major intrinsic proteins (MIP) that comprises the membrane channel proteins, which are selective for either water only (aquaporins) or water and other uncharged solutes, like glycerol and urea (aquaglyceroporins) [11].

The physiological role of Fps1 is the regulation of intracellular level of glycerol in response to changes in osmolarity [29]. In response to hyperosmotic stress Fps1 closes to allow glycerol accumulation in the cytosol and opens to release glycerol out of the cell under hypoosmotic conditions. Gating of Fps1 channel is mediated by the cytosolic N-terminal tail, and its truncation renders Fps1 constitutively open and allow an unregulated bidirectional transport of glycerol. Thus, yeast overexpressing of *fps1*-Δ*1* allele lacking N-terminal extension were hypersensitive to As(III) and Sb(III) and accumulated more As(III), while deletion of *FPS1* gene or high-osmolarity conditions profoundly increased resistance to As(III) and Sb(III) [28]. Interestingly, transport of metalloids via Fps1 is regulated at the transcriptional and post-transcriptional level. After As(III) and Sb(III) addition to the media, transcription of the *FPS1* gene is rapidly downregulated to reduce the Fps1 protein level [28]. Besides, activity of Fps1 is negatively regulated by the mitogen-activated protein kinase Hog1, which phosphorylates Fps1 at treonine 231 located in the N-terminal tail [30]. Deletion of *HOG1* or expression of Fps1-T231A resulted in increased sensitivity to As(III) and Sb(III) due to upregulation of metalloid intake. Thus, we proposed that As(III) and Sb(III) activate Hog1 kinase to phosphorylate N-terminal tail of Fps1 which results in channel closing and restriction of metalloid accumulation. Beese *et al*. [31] have shown that the pleckstrin homology (PH) domain proteins Rgc1 and Rgc2 positively regulate transport activity of Fps1 by yet unknown mechanism. Interestingly, deletion of *RGC1* and *RGC2* genes caused resistance to As(III) in both wild type and the *hog1*Δ mutant background suggesting that Hog1 may also effect activity of Fps1 by targeting Rgc1 and Rgc2. Indeed, As(III)-induced phosphorylation of Rgc2 was partially decreased in the absence of Hog1 but it has not been established whether Rgc2 is a substrate for Hog1.

A heterologous expression system using the yeast mutants lacking metalloid transporters, including Fps1, was successfully employed to identify plant aquaporins responsible for metalloid uptake [32–34]. Plant aquaporins are classified into four subfamilies corresponding to distinct subcellular localizations: small basic intrinsic proteins (SIP) located in the endoplasmic reticulum, plasma membrane intrinsic proteins (PIP) and tonoplast intrinsic proteins (TIP) responsible for water transport, and Nodulin26-like intrinsic membrane proteins (NIP), which are localized to plasma and intracellular membranes to mediate transport of NH_3_, B(OH)_3_ or Si(OH)_3_ [35–37]. Based on the plasma membrane localization and structural similarity between their physiological substrates and As(OH)_3_ or Sb(OH)_3_, NIPs were obvious candidates for testing their ability to transport As(III) and Sb(III) into the cells. Bienert *et al.* [32] have shown that *A. thaliana* AtNIP5;1, AtNIP6;1 and AtNIP7;1, *Oryza sativa* OsNIP2;1, OsNIP2;2 and OsNIP3;2, *Lotus japonicus* LjNIP5;1 and LjNIP6;1 increased sensitivity to As(III) and Sb(III) when expressed in *S. cerevisiae* lacking the aquaglyceroporin Fps1, thus highly resistant to metalloids. Importantly, decreased accumulation of As(III) in yeast cells expressing NIPs from *A. thaliana* and *O. sativa* was confirmed in direct transport studies [32]. Several other reports independently demonstrated a significant role of NIPs in As(III) uptake in *A. thaliana* (AtNIP1;1, AtNIP1;2, AtNIP5;2 and AtNIP7;1) and *O. sativa* (OsNIP1;1, Lsi1/OsNIP2;1, Lsi6/OsNIP2;2, OsNIP3;1) by showing their permeability to As(III) in *X. laevis* oocytes or increased As(III) tolerance and accumulation in the plant *nip* mutants [33,34,38]. In addition, the rice aquaporin Lsi1/OsNIP2;1 is also permeable to methylarsonic acid [MAs(V)] and dimethylarsinic acid [DMAs(V)] [39], while NIP1;1 in *Arabidopsis* contributes to Sb(III) uptake and sensitivity [40]. In sum, above findings suggest that all NIPs are permeable to As(III) and Sb(III) and some of them, like OsNIP1;1 and Lsi1/OsNIP2;1 in rice, constitute a major pathway for As(III) accumulation into roots and determine level of As(III) tolerance in plants (Figure 2). However, three members of rice PIP subfamily of aquaporins have been recently reported to mediate As(III) transport [41]. OsPIP2;4, OsPIP2;6, and OsPIP2;7 facilitated As(III) uptake in *X. laevis* oocytes, while overexpression of these proteins in *Arabidopsis* resulted in increased As(III) resistance. Moreover, the transgenic *Arabidopsis* lines expressing OsPIP2;6 exhibited both influx and efflux of As(III) depending on the outside concentration of this metalloid. This suggests that in plants both NIPs and PIPs are involved in As(III) transport down the concentration gradient (Figure 2).

It is believed that the active form of Sb(V)-based drugs used in the treatment of leishmaniasis is actually Sb(III), which is formed after reduction of Sb(V) inside macrophages. Thus, the role of aquaglyceroporins in metalloid uptake and tolerance was investigated in *Leishmania* parasites [42]. The *Leishmania major* AQP1 (LmAQP1), the closest homologue of human AQP9, has been shown to be responsible for As(III) and Sb(III) accumulation. Overexpression of *LmAQP1* gene in *L. tarentolae*, *L. infantum*, and *L. major* facilitated As(III) and Sb(III) uptake and resulted in hypersensitivity to both metalloids. Moreover, mutation in one of *LmAQP1* alleles in *L. major* increased resistance to Sb(III). Importantly, *LmAQP1* expression in wild type and antimony-resistant *L. donovani* strains could increase sensitivity to antimonials. Downregulation of AQP1 RNA level seems to be a major mechanism of antimony resistance found in field and clinical isolates of *Leishmania* [43,44].

Human genome encodes thirteen aquaporins (AQP0-12), out of which AQP3, AQP7, AQP9, and AQP10 are permeable for glycerol [45]. First, ability to transport As(III) and Sb(III) was demonstrated for two mammalian aquaglyceroporins, AQP7 and AQP9 (Figure 3) [46]. Expression of rat AQP9 in the yeast *fps1*Δ mutant reversed resistance to As(III) and Sb(III) and increased transport of As(III) and Sb(III) into the cells. Both mouse AQP7 and rat AQP9 were able to mediate As(III) uptake when expressed in *X. laevis* oocytes. Similarly, human aquaglyceroporins AQP7 and AQP9 were also shown to mediate As(III) uptake into oocytes, while no significant As(III) accumulation was observed when human AQP3 and AQP10 were expressed [47]. In this study, AQP9 proved to be the most efficient aquaglyceroporin in As(III) transport. Analysis of serial mutants of rat AQP9 in the pore region allowed to establish that As(OH)_3_ and glycerol use the same translocation pathway in AQP9 [47]. Based on the fact that AQP9 is primarily expressed in liver, spleen, testis and leukocytes [45], it has been proposed that AQP9 constitutes a major pathway for As(III) uptake from blood to liver, which plays a primary role in arsenic metabolism [45]. In addition, AQP9 contributes to As(III) sensitivity in leukemia cells [46]. Overexpression of AQP9, sensitized leukemia cells to metalloids, while As(III)-resistant leukemic cell lines displayed low level of AQP9 [48,49]. Analysis of aquaglyceroporin levels in an As(III)-resistant cell line (R15) derived from a human lung adenocarcinoma cell line (CL3) revealed that in the AQP7 and AQP9 non-expressing cells AQP3 may contribute to As(III) uptake and sensitivity [50]. Silencing of AQP10 expression with iRNA in the human colon adenocarcinoma Caco-2 cell line resulted in increased As(III) uptake, which suggests that AQP10 may be involved in intestinal absorption of As(III) [51]. Recently, involvement of seven aquaglyceroporins present in the zebrafish *Danio rerio* has been examined for metalloid transport [52]. Five of them, Aqp3, Aqp3l, Aqp9a, Aqp9b, and Aqp10, were able conduct both As(III) and Sb(III), with the exception of Aqp3, which was not permeable to Sb(III). Interestingly, both zebrafish and mammalian aquaglyceroporins were also shown to conduct organic methylated arsenicals, like methylarsonous acid [MAs(III)], MAs(V) and DMAs(V), however instead of the role in uptake, contributing to metalloid efflux and detoxification (see below) [52–54].

### 2.3. Role of Hexose Transporters in As(III) and Sb(III) Accumulation

Liu *et al.* [55] found that the yeast *fps1*Δ mutant accumulated As(III) at high rate when the transport experiments were performed in the absence of glucose. This observation led to the hypothesis that hexose transporters may be permeable to metalloids (Figure 1 and 3). Indeed, no accumulation of As(III) was observed in the yeast mutant devoid of all eighteen hexose transporter, whereas addition of glucose and other sugars inhibited As(III) uptake in wild type cells. Later, it has been shown that rat and human hexose transporter GLUT1, which belongs to the major facilitator superfamily (MFS), mediate As(III) accumulation when expressed in yeast cells and *X. laevis* oocytes [56]. The question was how glucose transporters could recognize As(III) as a substrate. It was suggested that three As(OH)_3_ molecules could polymerize to a ring structure that mimics the hexose molecule [55]. However, it seems to be unlikely in a view of the observation that GLUT1 can also catalyze transport of MAs(III), which did not compete with glucose transport [55]. This suggested that arsenicals and hexoses are differently translocated through GLUT1. Indeed, Jiang *et al.* [57] have shown that mutations in GLUT1 amino acid residues, which are critical for glucose uptake, increased the transport rate of MAs(III), while inhibitors of glucose transport did not affect MAs(III) flow. It has been speculated that arsenicals may use the water pathway for translocation via mammalian GLUT1, which is different from the glucose pathway [57]. Two more glucose permeases, GLUT2 in hepatocytes [58] and GLUT5 in the human colon adenocarcinoma Caco-2 cell line [51], were also reported to participate in uptake of As(III). Finally, OATPB [51] and OATPC [59], two human members of the organic anion transporting polypeptide family (OATP) involved in the transport of a wide range of amphipathic endogenous and exogenous organic compounds [60], were identified as the additional pathways for As(III) influx.

## 3. Transporters for As(III) and Sb(III) Detoxification

Metalloids accumulate very easily in the cells and all organisms developed several mechanisms to evade their toxicity in the cytosol. Once As(V) enters the cytoplasm, it undergoes a rapid enzymatic reduction to trivalent form, which is a substrate for multiple modes of modification and detoxification (Figures 1–3) [6,7]. In *S. cerevisiae*, As(V) reduction is mediated by the Acr2 arsenate reductase from the rhodanese/Cdc25 phosphatase superfamily [61]. Members of this family have been shown to mediate As(V) reduction also in *Leishmania* [62], plants [63,64] and humans [65]. Next, As(III) may be either extruded out of the cell by the As(III)-specific transporters of Acr3 family against the concentration gradient or via the aquaglyceroporins and the glucose permeases down the concentration gradient [6–8]. As(III) may be also sequestrated by glutathione (GSH) and phytochelatins (PCs), and the resulting complexes are the substrates of various ATP-binding cassette (ABC) transporters located in the vacuole and plasma membranes. In mammals, methylation of inorganic As(III) is a common phenomenon [58]. Methylated forms of arsenic are then exported from the cells by multiple ABC transporters, aquaglyceroporins and glucose permeases [51,54,58].

### 3.1. Involvement of Aquaglyceroporins in As(III) and Sb(III) Efflux

Aquaglyceroporins are bidirectional channels which mediate transport of substrates down the concentration gradient. Thus, in theory aquaglyceroporins could be involved not only in metalloid uptake but also in efflux contributing to metalloid tolerance under certain conditions. Indeed, the legume symbiont *Sinorhizobium meliloti* expresses an unusual arsenic resistance *ars* operon containing the aquaglyceroporin gene *aqpS*, instead of the arsenite transporter gene *arsB* [66]. AqpS confers resistance only to As(V) but exports As(III), which is formed by the action of arsenate reductase ArsC encoded by the same operon. Furthermore, two reports indicated that heterologous expression of several plant aquaglyceroporins in *S. cerevisiae* led to increase in As(V) resistance strongly suggesting that the aquaglyceroporins may play a conserved physiological role in trivalent metalloid efflux down the concentration gradient during As(V) poisoning [32,33]. Zhao *et al.* [67] have recently shown that the rice aquaglyceroporin Lsi1 (OsNIP2;1) facilitated extrusion of As(III) from *X. laevis* oocytes. When the *lsi2* rice mutant was exposed to As(V), level of As(III) markedly increased in roots, while As(III) efflux was reduced comparing to wild type plants. This strongly indicates that the plant aquaglyceroporins are capable of As(III) efflux under physiological conditions (Figure 2).

Consequently, we have demonstrated that the aquaglyceroporin Fps1 mediates As(III) efflux and is essential to maintain As(V) tolerance in budding yeast [68]. The *fps1*Δ mutant exhibited increase in sensitivity to As(V) and decreased As(III) extrusion out of the cell. Moreover, overexpression of both wild type Fps1 and the constitutively open variant Fps1-Δ1 led to higher As(V) tolerance. We have proposed that Fps1 functions as a bidirectional As(III) channel, which contribute to efflux of As(OH)_3_ produced by the arsenate reductase Acr2, when cells are exposed to As(V) (Figure 1). However, Fps1 overexpression rendered the cells more resistant also to As(III) and Sb(III), whereas prolonged As(III) treatment caused strong upregulation of Fps1 mRNA level, indicating that Fps1 may have an additional physiological function in metalloid efflux during As(III) exposure. How would it be possible in a view of the fact that the aquaglyceroporins are not able to transport solutes against the concentration gradient? Based on the observation of Markus J. Tamás group showing that yeast cells export GSH to chelate As(III) in the external medium and prevent in this way accumulation of As(III) into the cells [69], we have hypothesized that after longer exposure to As(III), the concentration of free As(OH)_3_ in the medium may considerably decrease and become lower than inside the cells, allowing efflux of intracellular As(OH)_3_ via Fps1 [68]. In support of this notion, identification of two GSH exporters from the MFS family, Gex1 and Gex2, has been recently reported in *S. cerevisiae* [70]. However, the role of these transporters in metalloid tolerance has not been investigated.

In mammals, mainly in liver, the intracellular arsenic is methylated in an multistep process catalyzed by the arsenic (+3 oxidation state) methyltransferase (AS3MT) resulting in the formation of MAs(V), MAs(III), DMAs(V), and DMAs(III), which are then excreted into bile and urine and cleared from the body [71]. Demonstration that the liver aquaglyceroporin AQP9 is capable of transport of methylated arsenicals, like MAs(V), MAs(III), and DMAs(V), interestingly at higher rate than As(OH)_3_, led to the conclusion that AQP9 is involved not only in As(OH)_3_ uptake from blood to liver but also in removal of methylated forms of arsenic down the concentration gradients from hepatocytes to the blood flow to end up in urine (Figure 3) [53,54]. The results of Carbrey *et al*. [72] showing that the AQP9-null mice are more sensitive to As(III), accumulate more As(III) in the body and excrete less arsenic species in the feces and urine, confirm a crucial role of aquaglyceroporins in arsenic detoxification in mammals. It is important to mention that the glucose permeases may act along with AQP9 as the exporters of methylated arsenicals along concentration gradients [51,56–58].

### 3.2. Acr3 Family of Metalloid Transporters

Microorganisms are able to withstand high concentrations of As(III) and Sb(III) thanks to the existence of metalloid-specific transporters which are located in the plasma membrane to facilitate immediate extrusion of these toxic compounds out of the cell [73]. Under non-stress conditions expression of genes encoding metalloid transporters is off but undergoes rapid induction in response to metalloid treatment leading to accumulation of efflux transporters in high amounts at the membranes. So far, two distinct families of transporters detoxifying As(III) and Sb(III) have been identified: the ArsB family, which is ubiquitously present in prokaryotes, and the Acr3 (arsenical resistance 3) family, whose members are found in bacteria, archaea, fungi (with the exception of fission yeast *Schizosaccharomyces pombe*) and recently found in genomes of lower plants [73–75]. ArsB transporters have twelve transmembrane spans and belong to the MFS superfamily. The *E. coli* ArsB acts as an As(OH)_3_/H^+^ and Sb(OH)_3_/H^+^ antiporter or as an ATP-dependent pump, when present in a complex with the catalytic subunit ArsA [73,76,77]. There are no obvious ArsB orthologues in eukaryotes. However, the silicon exporter Lsi2/OsNIP2;1 from rice, which is also permeable to As(III), shows weak homology to the *E. coli* ArsB (Figure 2) [34].

The *S. cerevisiae* Acr3 is the best characterized eukaryotic member of Acr3 family, which belongs to the bile/arsenite/riboflavin transporter (BART) superfamily [78]. The *ACR3* gene was isolated on a multicopy plasmid conferring resistance to high concentrations of As(III) in budding yeast [79]. Cells lacking Acr3 were hypersensitive to arsenicals and accumulated more As(III) comparing to wild type cells [80,81]. Based on these observations it has been concluded that Acr3 confers tolerance to arsenicals by mediating extrusion of As(III) out of the cell (Figure 1). Recently, we have shown that Acr3 is indeed localized to the yeast plasma membrane [82]. In addition, we have demonstrated that As(III) uptake into inside-out plasma membrane vesicles prepared from the Acr3-expressing yeast cells is coupled to the electrochemical potential gradient of protons generated by the plasma membrane H^+^-ATPase [83]. In contrast to ArsB, which is permable to both As(III) and Sb(III), members of Acr3 family seemed to be specific for As(III) transport. Acr3 from *Bacillus subtilis* [84], *Corynebacterium glutamicum* [74,85], *Alkaliphilus metalliredigens* [74], *S. cerevisiae* [79,80], and *S. douglasii* [86], have been reported to mediate only As(III) resistance and transport. However, the *Synechocystis* Acr3 mediates tolerance to Sb(III) and As(III) [87], while Acr3 in *Schewanella oneidensis* is involved in resistance to As(V) only [88]. Hence, we have analyzed the role of Acr3 in Sb(III) resistance in greater details and found that the *acr3*Δ mutant showed some sensitivity to Sb(III) and increased Sb(III) accumulation [82]. Although, Acr3 exhibited similar transport affinity for both metalloids, Acr3 transported As(III) three times faster than Sb(III) [83]. We believe that such properties of yeast Acr3 explain a minor role of this antiporter in Sb(III) tolerance.

However, little is known about molecular mechanisms of metalloid translocation via Acr3. Members of the Acr3 family exhibit ten-transmembrane topology [89] and lack any known sequence signatures, which could suggest mechanism of metalloid flow. Fu *et al.* [74] have revealed that a single cysteine residue (Cys129) in the *C. glutamicum* Acr3, which is located in the fourth transmembrane span and conserved in all members of the Acr3 family, is indispensable for As(III) efflux. We have found that the mutation of this highly conserved residue (Cys151Ala) in the *S. cerevisiae* Acr3 also led to a complete loss of transport activity [90]. This strongly suggests that interactions between As(III) and a thiol group of this residue is required to activate transport through Acr3. In addition, glutamate 305 in the *C. glutamicum* Acr3 is required for transport activity and is possibly involved in proton translocation during As(OH)_3_/H^+^ antiport [85].

Expression of the yeast Acr3 is mainly regulated at the transcriptional level. In response to As(III), As(V), and Sb(III) treatment, the AP-1-like transcription factor Yap8 is stabilized and activated by yet unknown mechanism to induce transcription of *ACR3* gene [91–93]. Many membrane transporters are also subjected to a post-translational control affecting protein stability and intracellular localization. However, we found no evidence that the presence of metalloids influences either turnover of Acr3 protein or its targeting to the plasma membrane [82].

Recently, Indriolo *et al.* [75] have shown that the arsenic hyperaccumulating fern *Pteris vittata* contains *ACR3* gene (*PvACR3*), which was able to complement As(III) sensitivity of the yeast mutant lacking As(III) detoxification system. Expression of *PvACR3* was strongly upregulated in fern gametophytes and sporophyte roots upon addition of As(III) to the external medium. Silencing of Acr3 transcripts in gametophytes caused an As(III) sensitive phenotype. In contrast to the *S. cerevisiae* Acr3, PvACR3 was shown to be localized to the vacuole membrane suggesting that in multicellular organisms Acr3 mediates sequestration of As(III) into the vacuole. Interestingly, *P. vittata* expresses two copies of *ACR3* gene which may contribute to the ability of this species to hyperaccumulate As(III).

### 3.3. Role of ABC Transporters in Metalloid Transport

In eukaryotes lacking metalloid-specific transporters, detoxification of As(III) and Sb(III) relies on metalloid sequestration by GSH and/or PCs and translocation of resulting complexes into the vacuole or out of the cell by members of two subfamilies of ABC transporters, multidrug resistance family (MDR or subfamily B, ABCB) and multidrug resistance-associated protein family (MRP or subfamily C, ABCC) [6,58,94]. ABC transporters are ubiquitously presents in prokaryotes and eukaryotes and constitute one of the most abundant family of membrane proteins. A typical ABC transporter consists of two membrane-spanning domains (MSD1 and MSD1) and two highly conserved nucleotide-binding domains (NBD1 and NBD2), which bind and hydrolyze ATP providing energy for transport. Some of MRP-type transporters are characterized by an additional N-terminal MSD0 domain. ABC transporters are involved in transport of a wide variety of substances, including membrane components, peptides, endogenous metabolites, xenobiotics and heavy metals [95,96].

Although, *S. cerevisiae* possesses the metalloid-specific exporter Acr3, detoxification of As(III) and Sb(III) is supported by two vacuolar ABC transporters, Ycf1 and Vmr1 (Figure 1) [81,91,97]. Ycf1 shows high similarity to human MRP1 and is involved in vacuolar sequestration of endogenous toxins and GSH-conjugated heavy metals [98]. Ycf1 was shown to mediate As(GS)_3_ transport, which was inhibited by the presence of Sb(III) [81]. Moreover, the *S. cerevisiae* cells lacking Ycf1 were moderately sensitive to As(III) and highly sensitive to Sb(III), while the double *acr3*Δ *ycf1*Δ mutant was the most sensitive to both metalloids [81,91]. Thus, the plasma membrane metalloid-specific antiporter Acr3 and the vacuolar ABC transporter Ycf1 constitute two distinct but complementary metalloid detoxification pathways in yeast. Vmr1 is another member of the MRP family and very close homologue of Ycf1. Wawrzycka *et al.* [97] have recently reported that deletion of *VMR1* gene in the yeast multiple mutant devoid of seven ABC transporters including Ycf1 as well as two transcription factors Pdr1 and Pdr3 caused increased sensitivity to cadmium and As(III). Since Vmr1 is localized to the vacuolar membrane, we speculate that Vmr1 may play a role in transport of GSH-conjugated As(III) into the vacuole.

Chelation of heavy metals and metalloids by glutathione-derived peptides called phytochelatins (PCs) constitutes a major detoxification mechanism in the fission yeast *S. pombe*, the nematode *Caenorhabditis elegans* and all plants [99]. Synthesis of PCs is catalyzed by the PC synthase (PCS), which is constitutively expressed but its activity is strongly increased in response to heavy metals and metalloids [100]. In the next step, the resulting metal(loid)-PC complexes are sequestrated in vacuoles. It was believed that the half-molecule ABC transporter Hmt1 is responsible for accumulation of PC-cadmium complexes into the vacuole of *S. pombe* [101]. However, it is important to mention that Hmt1 has no role in resistance to As(III) and Sb(III) in *S. pombe*, while plants have no homologues of HMT-1 [102,103]. On the other hand, the *C. elegans* HMT-1 was shown to confer tolerance not only to cadmium but also to arsenic and copper [104]. However, several studies have proved independently that HMT-1 transporters in *S. pombe*, *C. elegans*, and *Drosophila melanogaster*, are not required for PC sequestration in vacuoles [102–104]. Recently, Song *et al.* [105] have demonstrated that two *A. thaliana* ABC transporters, ABCC1 (MRP1) and ABCC2 (MRP2), mediate translocation of As(III)-PC complexes from the cytosol to the vacuole (Figure 2). Plants devoid of ABCC1 and ABCC2 became sensitive to arsenicals and displayed residual accumulation of As(III)-PC in vacuoles, whereas simultaneous overexpression of *AtABCC1* and the phytochelatin synthase gene *AtPCS1* resulted in As(III) resistance. In addition, heterologous expression of AtABCC1 or AtABCC2 in *S. cerevisiae* rendered the cells more resistant to As(III) but only when AtPCS1 was also expressed. These results have firmly established the role of ABCC-type transporters in facilitating the storage of As(III)-PC complexes in vacuoles as a major As(III) detoxification mechanism in higher plants. Similarly, the Ycf1 homologue in fission yeast, the ABCC2-type transporter Abc2 has been also shown to facilitate accumulation of cadmium-PC complexes into the vacuole [106].

In parasitic protozoan *Leishmania*, acquired resistance to antimonials is strongly associated with the overexpression of ABC transporter MRPA (ABCC3), formerly known as PGPA, which belongs to the MRP/ABCC subfamily [107–109]. MRPA mediates metalloid resistance by sequestrating As(III)- and Sb(III)-thiol conjugates into the intracellular vesicles [109]. Another member of the ABCC subfamily, PRP1 (ABCC7) has been demonstrated to confer resistance to pentamidine and Sb(III) when overexpressed in *L. major* [110]. It has been hypothesized that both MRPA and PRP1 contribute to accumulation of metalloids into the intracellular organelles, which are then subjected to exocytosis at the flagellar pocket to release their content out of the cell [109,111]. In the nematode *C. elegans*, three ABC transporters, *P*-gp1, *P*-gp3, and MRP1, were shown to confer resistance to As(III) [112]. Functional inactivation of *mrp-1* gene rendered animals hypersensitive to As(III) acute exposure, while deletion mutations in *pgp-1* and *pgp-3* genes caused moderate sensitivity. Increased resistance to arsenicals is also associated with expression of MRP-like genes in zebrafish [113,114]. High-level resistance to cadmium, As(III) and mercury observed in the zebrafish fibroblast-like cell line ZF4-Cd could be reversed by inhibitors of MRP-transport activity [113]. Moreover, As(III) induced transcription of *abcc1*/*mrp1*gene in the ZR4 cells and zebrafish embryos, while overexpression of this gene increased survival of zebrafish embryos in the presence of heavy metals and As(III) [114].

The *MDR1* (*ABCB1*) gene coding a *P*-glycoprotein was the first ABC transporter gene correlated with arsenic resistance in humans. Chin *et al.* [115] have shown that expression of *MDR1* is elevated in human renal carcinoma cells after As(III) treatment. Later, association of *P*-glycoprotein overproduction with development of cellular tolerance to As(III) was reported in the rat liver epithelial cell line, while addition of *P*-glycoprotein inhibitor could reverse resistance to As(III) [116]. Furthermore, the knockout mice lacking *MDR1* genes were more sensitive to As(III) than wild type and accumulated more As(III) in organs [117,118]. This indirectly suggested that mammalian *P*-glycoprotein is involved in As(III) efflux (Figure 3). However, since *P*-glycoprotein does not transport glutathione conjugates, more studies are needed to establish mechanism of As(III) transport by this class of proteins.

In humans, there are nine members of MRP/ABCC subfamily, MRP1-MRP9, of which MRP1 and MRP2 are well-characterized players in As(III) detoxification system (Figure 3) [94]. The involvement of remaining five MRPs in metalloid tolerance remains to be established. First, it was observed that overexpression of MRP1 and/or MRP2 in cultured mammalian cell lines led to increased resistance to arsenicals and antimonials, which was associated with MRP-dependent metalloid efflux [50,116,119–122]. Moreover, cell lines lacking functional *MRP1* genes exhibited higher sensitivity to As(III) [123,124]. Direct transport studies have revealed that human MRP1 facilitates efflux of inorganic As(III) and methylated MAs(III) species conjugated with GSH [125,126]. Based on the analysis of arsenic species found in the bile of wild type and MRP2-deficient rats, it was concluded that MRP2 is responsible for biliary excretion of As(III) and MAs(III) in GSH-dependent way [127]. In a recent study, using membrane vesicles prepared from human embryonic kidney cells (HEK293T) transfected with human MRP2, Carew and Leslie [128] have shown that in addition to As(GS)_3_ MRP2 mediates transport of [(GS)_2_AsSe]^−^. Since MRP2 is the only MRP-like ABC transporter localized to the apical surface of epithelial cells in liver, it has been proposed that the physiological role of MRP2 in metalloid detoxification is to excrete arsenicals into bile and then in feces [94,128]. Both MRP1 and MRP2, and other MRP proteins, are expressed in various organs and may certainly contribute to metalloid detoxification throughout the human body [128].

## 4. Perspectives

Studies on efflux and uptake routes of arsenic and antimony, including identification of metalloid transport proteins, regulation of their expression and control of metalloid transport, is crucial for understanding of metalloid tolerance mechanisms. Such knowledge can be used to sensitize target cells to metalloid-based drugs in anticancer and antiprotozoan therapies as well as to increase arsenic accumulation in species used for phytoremediation or on the other hand to downregulate metalloid intake in normal cells and crops. For example, it has been recently reported that the *S. cerevisiae ACR3* gene can be successfully expressed in higher plants [129]. Introduction of the yeast *ACR3* gene into rice resulted in significant reduction of As(III) accumulation in shoots and roots due to active extrusion of arsenic from root cells to the external medium. Thus, cultivation of *ACR3*-expressing transgenic lines could lower arsenic accumulation in crops grown in areas contaminated by metalloids. Moreover, targeting the fern As(III) transporter Acr3 to the vacuolar membrane in higher plants could lead to development of new species suitable for arsenic phytoremediation in various geographic zones. Another example is the construction of transgenic *A. thaliana* which accumulates two-ten-fold more As(III) and two-three-fold more As(V) than wild-type plants as a result of the simultaneous overexpression of the garlic phytochelatin synthase *AsPCS1* and the yeast vacuolar ABC transporter *YCF1* genes [130].

As much progress has been made in identification of metalloid transporters in a number of eukaryotic organisms, future studies should be aimed at determining substrate specificity of these proteins, mechanisms of metalloid translocation across membranes and how activity of these transporters is regulated at the post-translational level. This would allow to search for new strategies for manipulation of metalloid transport across membranes to increase or decrease intracellular concentration of metalloids.
